# Evaluation of the Effectiveness of the Socket Preservation Technique Using Allogeneic and Xenogeneic Materials—A Preliminary Study

**DOI:** 10.3390/jfb16060192

**Published:** 2025-05-23

**Authors:** Piotr Wróbel, Adam Piecuch, Michał Bąk, Paweł Krynicki, Jakub Adamczyk, Piotr Mojżesz, Agnieszka Kiełboń, Sylwia Wójcik, Martin Starosta, Won-Pyo Lee, Tadeusz Morawiec

**Affiliations:** 1 Department of Oral Surgery, Faculty of Medical Sciences in Zabrze, Medical University of Silesia, 15 Poniatowskiego Street, 40-055 Katowice, Poland; pawel.krynicki@sum.edu.pl (P.K.); jakub.adamczyk@sum.edu.pl (J.A.); piotr.mojzesz@sum.edu.pl (P.M.); swojcik@sum.edu.pl (S.W.); tmorawiec@sum.edu.pl (T.M.); 2 Private Dental Practice Comfortmed, 12 Wspólna Street, 44-240 Żory, Poland; mbak@sum.edu.pl; 3 Department of Histology and Cell Pathology, Faculty of Medical Sciences in Zabrze, Medical University of Silesia in Katowice, 19 Jordana Str., 41-808 Zabrze, Poland; apiecuch@sum.edu.pl; 4 Department of Children’s Maxillofacial Surgery, Faculty of Medical Sciences in Katowice, Medical University of Silesia, 15 Poniatowskiego Street, 40-055 Katowice, Poland; 5 Faculty of Science and Technology, University of Silesia, 75 Pułku Piechoty 1A, 41-500 Chorzów, Poland; agnieszka.kielbon@us.edu.pl; 6 Department of Dentistry, Faculty of Medicine, University of Ostrava, Syllabova 19, 703 00 Ostrava-jih, Czech Republic; martin.starosta@osu.cz; 7 Department of Periodontology, School of Dentistry, Chosun University, Gwangju 61452, Republic of Korea; wplee8@chosun.ac.kr

**Keywords:** bone augmentation, biomaterial, socket preservation, extraction, deproteinized bovine bone, allograft

## Abstract

Background: The socket preservation technique involves filling the bone defect created after tooth extraction with a bone substitute material. This helps to reduce bone resorption of the post-extraction alveolar ridge. Various types of bone substitute biomaterials are used as augmentation materials, including autogeneic, allogeneic, and xenogeneic materials. The purpose of this study was to evaluate changes in alveolar ridge dimensions and alterations of optical bone density in sockets grafted with two different biomaterials. Additionally, bone biopsies taken from the grafted sites underwent histological evaluation. Methods: This study enrolled 10 generally healthy patients, who were divided into two equal groups. Patients in the first group were treated with an allogeneic material (BIOBank^®^, Biobank, Paris, France), while patients in the second group were treated with an xenogeneic material (Geistlich Bio-Oss^®^, Geistlich Pharma AG, Wolhusen, Switzerland). Tooth extraction was performed, following which the appropriate material was placed into the debrided socket. The material was secured with a collagen membrane (Geistlich Bio-Gide^®^, Geistlich Pharma AG, Wolhusen, Switzerland) and sutures, which were removed 7 to 10 days after the procedure. Micro-CBCT examinations were performed, for the evaluation of alveolar ridge dimensions and bone optical density, at 7–10 days and six months after the procedure. Bone trepanbiopsy was performed simultaneously to the implant placement, six months after socket preservation. The retrieved biopsy was subjected to histological examination via hematoxylin and eosin (H&E) staining and Masson’s trichrome staining. Results: The results showed that the allogeneic material was more effective in preserving alveolar buccal height and was probably more rapidly transformed into the patient’s own bone. Sockets grafted with the xenogeneic material presented higher optical bone density after six months. Both materials presented similar effectiveness in alveolar width preservation. Conclusions: Based on the outcomes of this study, it can be concluded that both materials are suitable for the socket preservation technique. However, the dimensional changes in the alveolar ridge and the quality of the newly formed bone may vary depending on the type of biomaterial used.

## 1. Introduction

Tooth extraction is one of the most commonly performed procedures in dentistry. The direct consequence of the procedure is the creation of a bone defect filled with a blood clot. The healing of the socket is a multi-stage process lasting many months or even years [1]. During this time, there is a reduction in the vertical and horizontal dimensions of the alveolar ridge, which is most pronounced in the first six months after extraction [2,3]. The described volume reduction adversely affects planned prosthetic or implantoprosthetic treatments.

The vast majority of studies have confirmed that the use of an alveolar ridge preservation technique, consisting of filling the socket with an augmentation material, reduces vertical and horizontal alveolar atrophy [4,5,6,7,8]. The material inserted into the socket provides a scaffold that helps stabilize and mature the blood clot in the first stages of healing and promotes the formation of new bone by osteoblasts in the later stages through its osteoconductive effect [1,9].

Many different types of materials can be used for socket preservation, such as autogenous bone, allogeneic bone, xenogeneic bone, alloplastic materials, collagen cones, PRF, and autologous dentin matrix [8,10,11,12,13,14,15]. Each of these materials has different properties, which include osteoconduction, osteoinduction, and osteogenesis.

Osteoconduction is the ability of a material to form a scaffold for newly forming bone tissue. Osteoinduction is the ability of a biomaterial to stimulate undifferentiated mesenchymal cells to differentiate into osteoblasts, thereby stimulating bone formation. Osteogenesis is the ability of living cells in the augmentation material to produce new bone.

Autologous bone is considered to be the “gold standard” of graft materials. It is the only material that has a capacity for osteogenesis, osteoinduction, and osteoconduction. Autografts carry no risk of cross-infection or immune response [16]. The need for surgical access to the donor site, the limited availability of materials, and its faster resorption compared to other biomaterials make autogenous bone less commonly used in alveolar ridge preservation procedures [17,18]. It is common practice to mix autologous bone with other materials to combine the properties of both, improving the volumetric stability of the graft [19].

Alloplastic materials are synthetic materials used for bone grafting. This broad group includes hydroxyapatite (HA), beta-tricalcium phosphate (β-TCP), calcium sulfate, octacalcium phosphate, bioactive glass, and polymers such as PMMA or HEMA, among others [20,21,22,23]. These materials show great heterogeneity in terms of properties such as their volumetric stability, porosity, cytotoxicity, and mechanical characteristics; however, they are all only osteoconductive [20,22,23]. Their advantages are low immunogenicity, unlimited availability, and no risk of cross-infection, while their disadvantages include a lack of osteoinduction and osteogenesis and a theoretical risk of a immune response [16,24]. Their variable mechanical and biological properties can be both an advantage and a disadvantage.

Xenografts are materials of animal origin which exhibit an only osteoconductive ability. They are made from bone that has undergone extensive processing to remove its organic components and reduce immunogenicity, leaving a mineral scaffold primarily composed of hydroxyapatite [20]. While materials of bovine origin (e.g., Bio-Oss^®^) are most commonly used, substitutes derived from equine, porcine, algal, or coral species are also available. The advantages of xenografts include their unlimited availability, good volumetric stability due to their slow resorption, and how similar their porosity is to that of human bone, while their disadvantages include a lack of osteogenesis, a lack of or weak osteoinduction ability, a risk of immune response, a risk of zoonosis transmission, and ethical and religious issues [16,20,22].

Allogeneic bone grafts are obtained from living donors or cadavers and processed through methods such as freeze-drying (FDBA) or demineralization (DFDBA) to remove immunogenic components while preserving the mineral and collagen matrix [23,24]. Freeze-dried bone allografts only have osteoconductive properties, whereas demineralized freeze-dried bone allografts have both osteoconductive and osteoinductive properties due to the exposure of their bone morphogenetic proteins (BMPs) during the demineralization process [25,26,27]. Removal of the mineral component reduces the volumetric stability of the graft [24,28,29]. To achieve osteoinductive capacity while maintaining volumetric stability, the partial demineralization of the allograft can be performed, yielding a material with intermediate characteristics, such as BIOBank^®^ [28]. As with xenografts, the availability of allografts is unlimited, but there is a risk of immune reaction or cross-infection [16,23,24]. Ethical and religious issues also need to be considered.

The main aim of this study was to compare the effectiveness of xenogeneic versus allogeneic materials in alveolar ridge preservation when used in combination with a collagen membrane. An additional objective was the histological examination of bone biopsies obtained during implant placement.

## 2. Materials and Methods

### 2.1. Patient Selection and Allocation

This study was conducted on 10 patients in which alveolar ridge preservation was performed following first or second maxillary premolar extraction. The patients were randomly divided into two groups according to the augmentation material used using the Research Randomizer software (https://www.randomizer.org) (accessed on 1 August 2024). The material used was partially demineralized bone matrix allograft (BIOBank^®^, cortico-cancellous bone powder, 0.5 mm granules) in group number 1 and deproteinized bovine bone matrix (DBBM) cancellous xenograft (Geistlich Bio-Oss^®^, 0.25–1 mm granules) in group number 2. In every patient, a collagen membrane (Geistlich Bio-Gide^®^) was used.

The inclusion criteria were as follows: an unrestorable first or second maxillary premolar scheduled for extraction, an age between 18 and 65 years, and being healthy or having well-controlled systemic disease that does not affect bone or soft tissue healing. Meanwhile, the exclusion criteria included smoking, alcoholism, immunosuppression, anticoagulant therapy, history of chemotherapy or radiation therapy of the head and neck in the last 5 years, history of antiresorptive therapy, extensive periapical lesion, poor oral hygiene, extensive tooth loss, non-controlled periodontitis, and alveolar buccal bone deficiency.

All of the participants were informed about the purpose and methods of this study, and their consent was obtained. The approval of the local ethic board was obtained for this study (approval number: BNW/NWN/0052/KB1/43/24; date: 21 May 2024).

The clinical part of this study was conducted at the “Comfortmed” dental clinic in Żory and the Department of Oral Surgery of the Medical University of Silesia in Bytom.

### 2.2. Extraction with Socket Preservation

**Figure 1:**
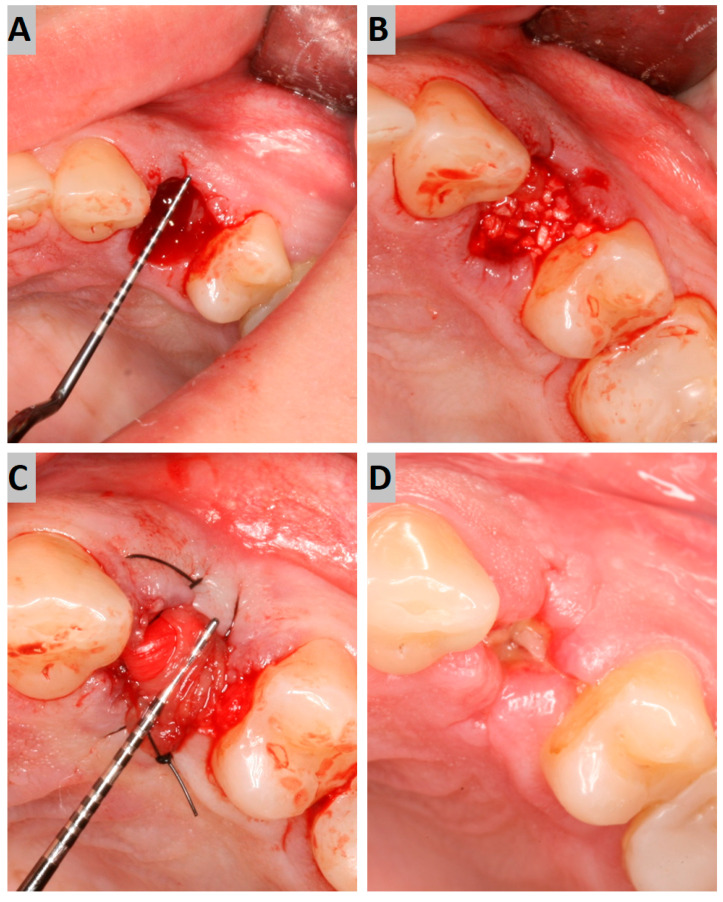
(**A**) Post-extraction socket; (**B**) augmentation with a grafting material; (**C**) collagen membrane suturing; (**D**) secondary intention wound healing after 7 days.

The atraumatic intra-alveolar extraction of the premolar was carried out under local anesthesia. The socket was then debrided of soft tissues and rinsed with sterile saline. The bony defect was filled with an appropriate augmentation material to the alveolar crest level (Figure 1A,B). On the buccal and palatal side, the minimal full-thickness envelope flaps were elevated to properly place the collagen membrane. The alveolus filled with augmentation material was then covered with the collagen membrane, which was placed subperiosteally and stabilized using 4–0 nylon sutures (Figure 1C). The surgical site was photographed while preserving the patients’ anonymity.

The post-surgical recommendations included oral antibiotics—namely, 1 g of amoxicillin or 600 mg of clindamycin in allergic patients—every 12 h for 7 days, topical chlorhexidine gel (Elugel) 3 times a day, and NSAIDs on demand.

The follow-up appointment was carried out 7 to 10 days after the surgery. The surgical site was photographed, sutures were removed, and CBCT with a 5 × 5 cm FOV and a voxel size of 75 µm (Carestream CS 8100 3D) was performed (Figure 1D).

### 2.3. Implant Surgery and Prosthetic Protocol

**Figure 2:**
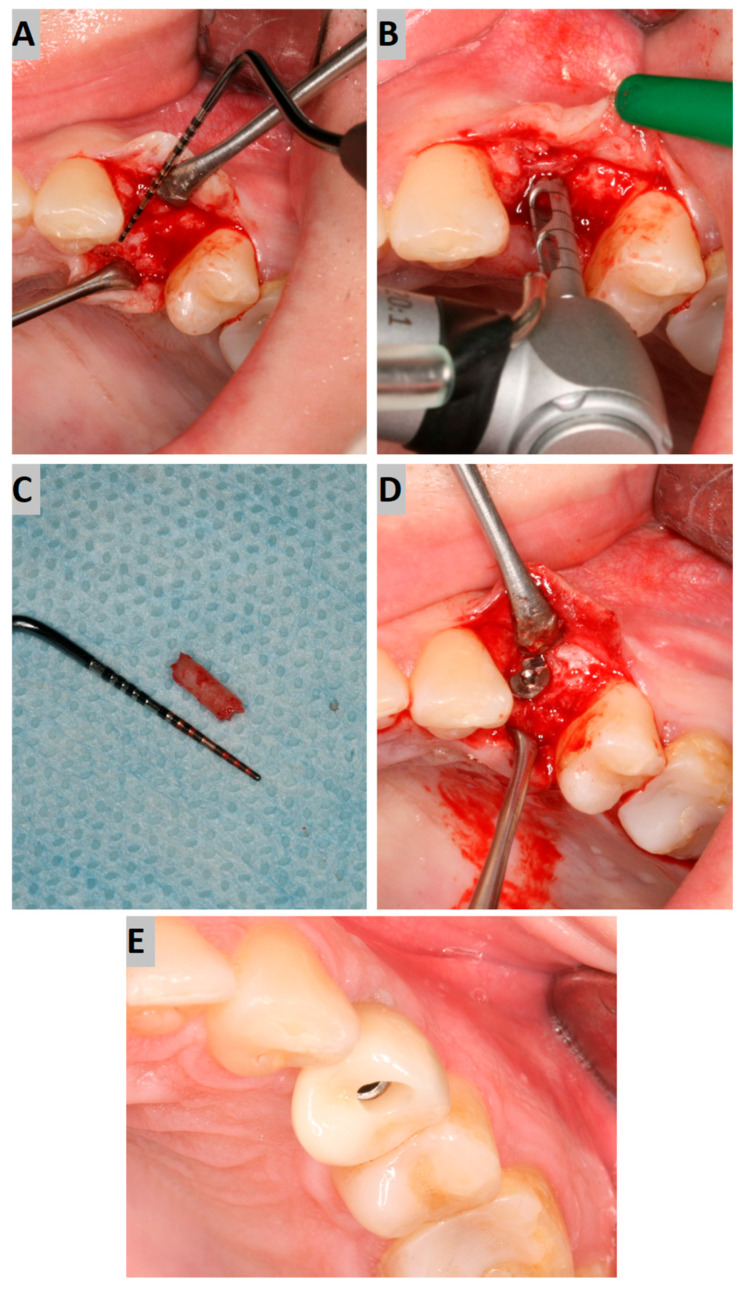
(**A**) Alveolar ridge after 6 months of healing; (**B**) bone biopsy with trephine; (**C**) obtained trepanobiopsy; (**D**) implant secured with cover screw; (**E**) screw-retained crown restoration.

Six months after the socket preservation procedure, another CBCT with a 5 × 5 cm FOV was performed to plan the placement of the implant in the augmented site (Figure 2A).

Implant placement was carried out, according to clinical conditions, either flaplessly with the aid of a tissue punch or conventionally with mucoperiosteal flap elevation. During the surgery, a trephine bone biopsy was performed using a trephine with a 2 mm inner diameter (Figure 2B,C). Next, the implant bed was prepared according to the drill sequence, and a dental implant of an adequate length and diameter (AB Dental I5) was placed, obtaining sufficient primary stability (>35 Ncm). Afterward, the cover screw was tightened to the implant, and the submerged healing protocol was employed (Figure 2D).

After another six months, implant uncovering was performed, resulting in healing abutment installation. Following 2 weeks of soft tissue healing, the scanbody was installed, and an intraoral scan was performed using an intraoral device (Dexis IS 3800 W). Then, the crown was fabricated. The screw-retained crown was adjusted occlusally and delivered to the patient (Figure 2E).

### 2.4. Radiographic Analysis

**Figure 3:**
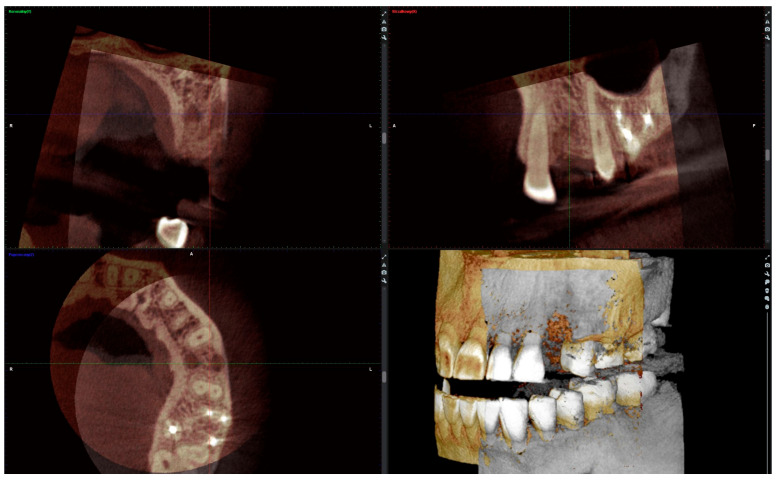
Superimposition of radiographic images.

**Figure 4:**
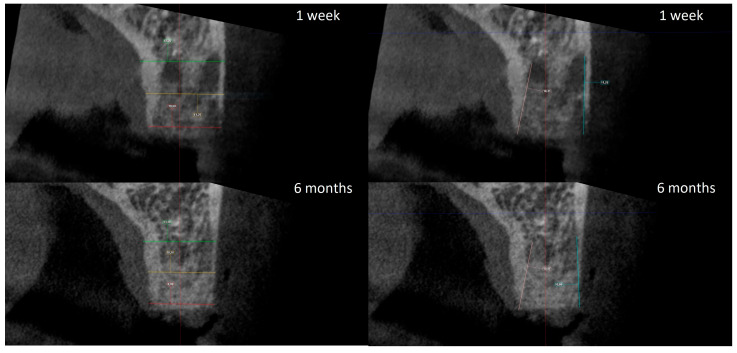
(**A**) Alveolar ridge width measurements: coronally (red line), midpoint (yellow line) and apically (green line); (**B**) alveolar ridge height measurements: buccally (blue line) and palatally (pink line).

The radiographic analysis consisted of alveolar ridge dimension measurements taken 1 week and 6 months post socket preservation surgery. The measurements were made using the Romexis software provided by the Planmeca company. The superimposition of the radiographic images was created automatically by selecting 3 reference points, most often the incisal edges and cusps of adjacent teeth. Afterward, the images were manually corrected (Figure 3). The above-mentioned steps ensured the repeatability of the measurements carried out on sagittal planes, which were oriented in such a manner that the buccal bone plate was parallel to the long axis. The measurements were made in the central part of the alveolus. The distance between the buccal and palatal bony plates was measured at the alveolar crest, the bottom of the alveolus, and midway between these two points (Figure 4A). The height was measured from the bottom of the alveolus to the top of the buccal and palatal bony plates, respectively (Figure 4B).

**Figure 5:**
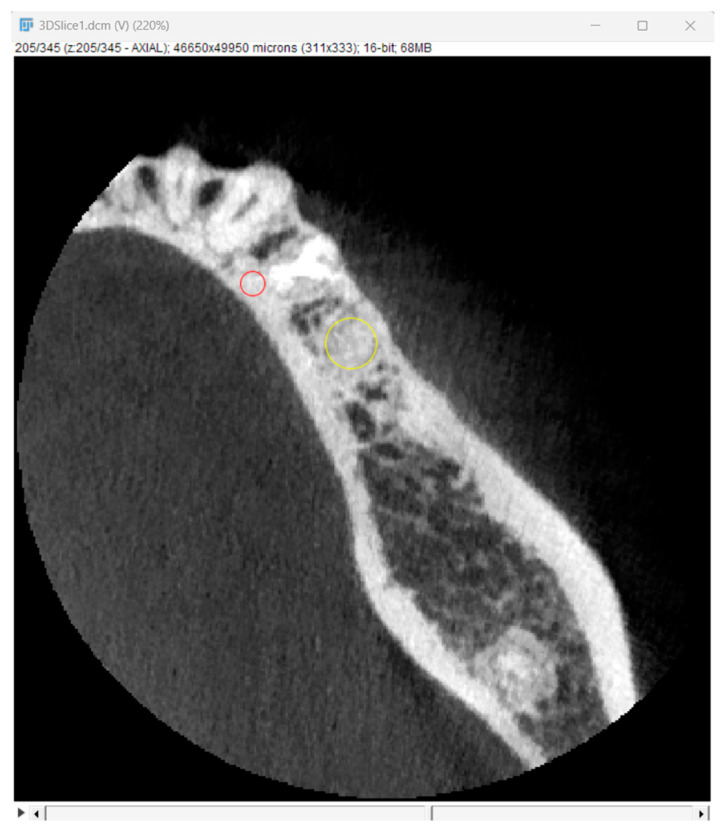
Radiographic optical density of bone measured using ImageJ software. The yellow circle represents the ROI where the measurements were taken, and the red circle represents the positioning pin used for orientation during the measurements.

Bone optical density was measured in Hounsfield units (HU) using ImageJ software (version 1.53k, Wayne Rasband and contributors, National Institutes of Health, Kensington, MD, USA). The readings were taken on the horizontal plane in the central part of the alveoli 7 days and 6 months after surgery (Figure 5). If an interradicular septum was present in the central part of the socket, measurements were taken more coronally to reduce the risk of bias.

### 2.5. Histological Analysis

Subsequent to their acquisition, bone trephine biopsies, intended for histochemical analysis, were stored and fixed in 4% stabilized buffered formaldehyde solution. Next, the specimens were transported to the Department of Histology and Cell Pathology, Medical University of Silesia. The fixation time ranged from 24 to 48 h. Excess fixative was removed during 40 min of rinsing under running water. Afterward, the specimens were stored in a 12% EDTA solution for 7 days. Following this procedure, the specimens were subjected to dehydration in a series of alcohol solutions with progressive concentrations ranging from 50% to 99.8%. The next step included processing the material through intermediary fluids, beginning with a 1:1 mixture of absolute alcohol and xylene (not longer than 15 min), followed by processing in pure xylene (2 × 10 min) to remove the remaining alcohol. In the next step, the tissues were placed in a 1:1 mixture of paraffin wax and xylene (for no longer than 20 min) to partially remove the xylene and initially imbue the tissue with paraffin wax. In the last step, the material was placed in pure paraffin wax to remove the remaining xylene and fully saturate the tissue before the material was finally embedded in a paraffin wax block.

Every paraffin block was sectioned using a rotary microtome (LEICA RM 2145) into 5 µm slices. Stretched slices were transferred to glass slides (Polysine Slides Microscope Slides, Menzel-Glaser, Stuttgart, Greece). Afterward, the slices were dewaxed and subsequently stained with hematoxylin and eosin and Masson’s trichrome stain (BIO 04-010802, Bio Optica, Santiago, Chile). The Masson method stains non-mineralized collagen blue and mineralized collagen red. This makes it possible to distinguish between woven bone and mature, more mineralized lamellar bone. The histochemical staining analysis was performed using the Eclipse 80i (Nikon, Kyoto, Japan) light microscope.

### 2.6. Statistical Analysis

The obtained radiological alveolar width and height measurements were subjected to statistical analysis using STATISTICA 13.1 (TIBCO Software Inc., Palo Alto, CA, USA) software. The differences between 5 parameters (width at crest, at midline, and at the bottom of the alveolus and buccal and palatal height) over 6 months of healing were compared between groups. The Shapiro–Wilk test was employed to check the normality of the distribution in both groups, and Levene’s test to check the equality of variances. All of the variables showed normal distribution and equality of variances. Therefore, an independent t-test was used to check the difference between the means of the two groups. In this manner, a comparison was made as to whether there were statistically significant differences between the measured parameters for the human-derived and animal-derived materials.

## 3. Results

### 3.1. Alveolar Ridge Radiographic Evaluation

The width and height measurements taken seven days and six months after surgery allowed for the calculation of the alterations of the given dimensions over the healing period. The obtained data are presented in Table 1.

The statistical analysis demonstrated no statistically significant differences between groups for all parameters except buccal height alteration. The latter was significantly higher in the xenograft group, with a 1.3 mm average, compared to the 0.5 mm average in the allograft group. For this particular comparison, the statistical significance was estimated to be *p* = 0.01.

### 3.2. Optical Bone Density Measurement

The optical density measurements were performed on 10 consecutive cross-sections selected from the central portion of the alveolus in each patient. The measurements were made for representative regions of interest (ROIs) in the augmented site. The data obtained are presented in Table 2.

It is worth noting that in the allograft group, the mean optical density increased from 1341.40 to 1499.94 HU. However, the difference was statistically insignificant; therefore, one may only conclude that there was a growth tendency. In contrast, in the xenograft group, the mean optical density increased from 1288.58 to 1881.08 HU, which was statistically significant at *p* = 0.004.

### 3.3. Histological Evaluation

**Figure 6:**
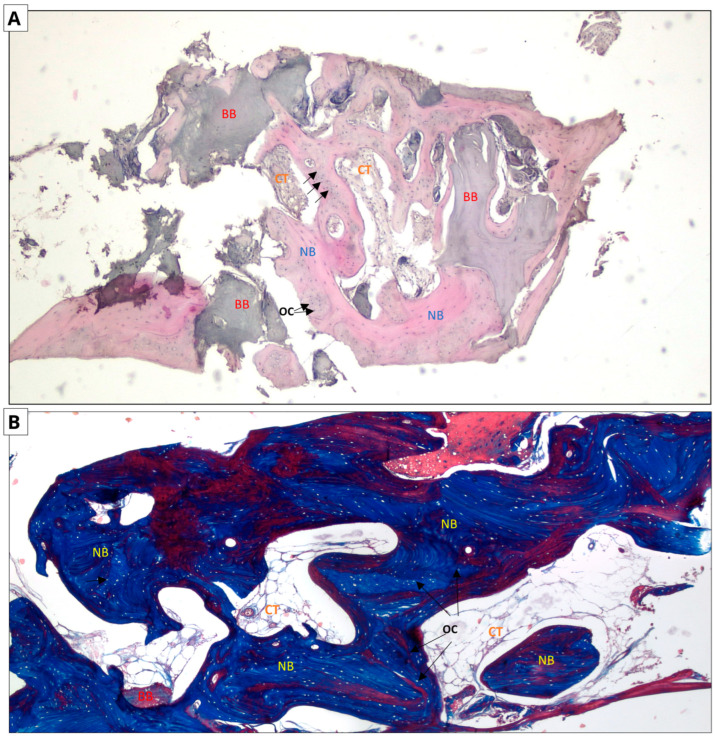
Allograft group. Two-dimensional (2D) reconstruction of the bone sample at ×40 magnification. (**A**) Hematoxylin and eosin staining; (**B**) Masson’s trichrome staining. Woven bone stained blue and lamellar bone stained red. Residual allograft particles (BB) surrounded by newly formed bone tissue (NB) containing osteocytes (OCs, arrows). Connective tissue (CT) consisting of fibroblasts and adipocytes.

**Figure 7:**
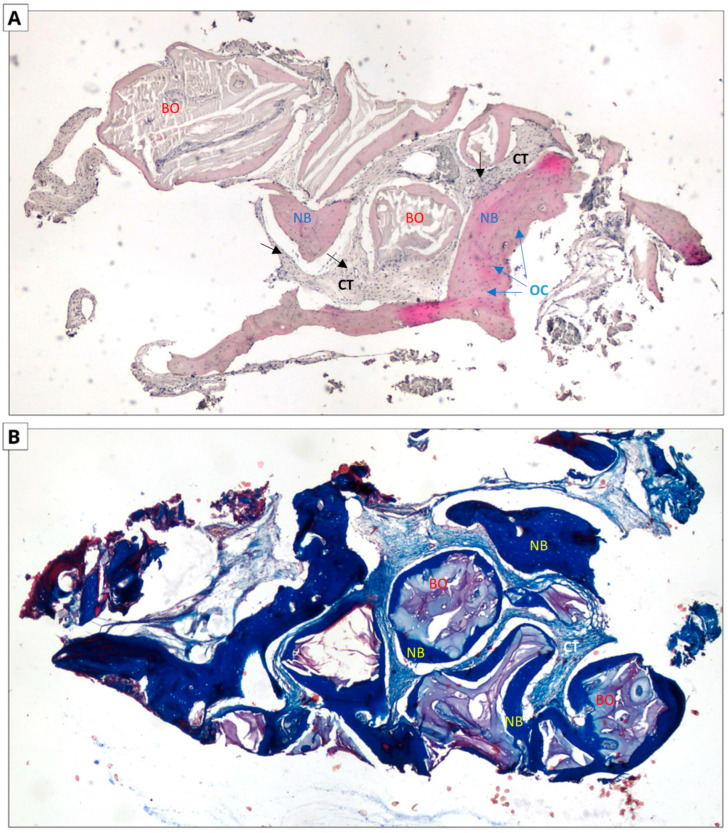
Xenograft group. Two-dimensional (2D) reconstruction of the bone sample at ×40 magnification. (**A**) Hematoxylin and eosin staining; (**B**) Masson’s trichrome staining. Woven bone stained blue and lamellar bone stained red. Residual xenograft particles (BO) surrounded by newly formed bone tissue (NB) with osteocytes (OCs, blue arrows). Connective tissue (CTs black arrows) consisting of fibroblasts and adipocytes.

The following micrographs show specimens stained with hematoxylin and eosin and Masson’s trichrome with aniline blue (Figure 6 and Figure 7). In all five trepanobiopsies, taken from patients with xenografts, residual graft material particles were found in the slides. In the group of patients where the augmentation material was an allograft, residual graft particles were found in only one trepanobiopsy.

In the case of hematoxylin and eosin staining, the presence of new bone tissue in direct contact with the augmentation material was observed in both samples. A similar structure to the connective tissue, with numerous fibroblasts and fewer adipocytes, was also visible, which may correspond to bone marrow. No inflammatory cells were observed. The above-mentioned observations indicate the biocompatibility of both materials.

In the case of Masson staining, some differences were observed. The samples from the xenograft group included areas of bone with an irregular arrangement of collagen fibers, stained blue, which may correspond to woven bone. In the samples from the allograft group, areas that may correspond to woven bone were also observed. In addition, there were noticeable areas of bone with a regular arrangement of collagen fibers, stained red, which may correspond to lamellar bone, indicating the completion of the bone remodeling process.

## 4. Discussion

The aim of this study was to compare the clinical effectiveness of xenogeneic and allogeneic materials in socket preservation. We presented the results obtained from the analysis of 10 cases. Due to the small research group, this study should be taken as a preliminary study. The obtained results suggest a comparable effectiveness of both materials regarding alveolar width preservation and the higher effectiveness of allografts regarding buccal bone height preservation. The failure to detect any allograft particles in the majority of the tested specimens led to the abandonment of the histomorphometric analysis. These findings suggest a faster remodeling of allografts into native bone. However, this should be interpreted cautiously due to a possible discrepancy between the optimal implant bed position and the augmented socket area.

An important factor influencing new bone formation is the use of barrier membranes. These membranes serve as a mechanical barrier against epithelial tissue ingrowth into bony defects, which secures sufficient time for bone formation [30]. The application of membranes improves the clinical effectiveness of alveolar ridge preservation [7,31]. In the present study, a collagen membrane was used in an open healing protocol, with minimal envelope flap preparation, providing mechanical protection for the graft particles and preventing soft tissue ingrowth into the augmented area. Such an approach minimizes the bone resorption of the alveolar ridge [32].

Abellán et al. compared the effectiveness of mineralized cortico-cancellous FDBA (MinerOss^®^, BioHorizons, Birmingham, AL, USA) and xenogeneic cancellous DBBM materials (Bio-Oss^®^) in socket preservation after five months of healing following a maxillary and mandibular molar extraction [33]. The allograft showed a tendency to reduce less alveolar width loss, whereas the xenograft showed a tendency to reduce alveolar height loss. The obtained results were statistically insignificant. A histological analysis showed that the ratio of residual graft to newly formed bone and connective tissue was similar between the groups. Compared to our study, the researchers used a non-demineralized allograft, which could explain the different results, and especially the presence of residual graft material in the allograft group.

Serrano Méndez et al. compared the effectiveness of demineralized freeze-dried cortical allografts (600–850 mm) and xenogeneic cancellous DBBM materials (Bio-Oss Collagen^®^, 250–1000 mm) in socket preservation after single-rooted tooth extraction [34]. The healing period was six months. The allograft group presented a tendency to lower alveolar width and height loss. Moreover, a histological examination revealed that the allografts had a higher tendency to remain in the form of residual particles. None of the results were statistically significant. The authors used demineralized cortical allografts. While the demineralization process accelerates the resorption of the biomaterial, the cortical nature of the graft may explain the higher tendency of the allograft to remain in the socket and provide better volumetric stability.

Sadeghi et al. showed a similar loss of vertical and horizontal alveolar dimensions with xenograft cancellous DBBM (Bio-Oss^®^) and cortico-cancellous DFDBA (CenoBone^®^, Cenobiologics Ltd., Milton Keynes, UK) after a healing period of four to six months [35]. Histological examination of the trephine biopsy showed a statistically significantly higher content of residual xenograft biomaterial particles. This explains the lower percentage of residual graft seen. The demineralization process increases material resorption and decreases volumetric stability, which may explain why the partially demineralized allograft used in our study showed less vertical buccal alveolar height loss.

Zampara et al. performed a histological analysis of trephine biopsies taken from sites after socket preservation using alloplastic (biphasic calcium sulfate, Bondbone^®^, MIS Implants Technologies, Shlomi, Israel), allogeneic (cancellous FDBA), and xenogeneic (cancellous DBBM, Bio-Oss^®^) materials [36]. The biopsies were taken after three months of healing. The results obtained, similar to our observations, showed a statistically significantly lower content of residual biomaterial particles in the samples from the allograft and alloplast groups compared to the xenograft group. Both xenograft and allograft materials were cancellous and mineralized in nature; therefore, the lower content of residual particles in the allograft group could be due to the different origin of the materials.

A meta-analysis of studies by Natto et al. on the effectiveness of alveolar preservation using xenogeneic and allogeneic materials showed that xenogeneic materials were more effective in maintaining alveolar width, whereas allogeneic materials were more effective in maintaining alveolar height [37]. However, these results were not statistically significant.

The results of alveolar ridge preservation may depend on many factors, not only the properties of the material used, but the studies that have addressed this issue are inconclusive and indicate the need for further research.

Leblebicioglu et al. showed that factors such as the condition of the marginal periodontium, the thickness of the vestibular lamina of the bone, and the initial width of the alveolus can affect the dimension alterations of the alveolar process after a socket preservation procedure [38]. These studies did not show a relationship between the structure of the bone tissue in the operated area and various clinical parameters. A relationship between the decrease in vestibular lamina thickness and greater alveolar resorption after alveolar preservation procedures was also shown by Avila-Ortiz et al. [39].

In contrast to the above-mentioned studies, Cardaropoli et al. and Spinato et al. did not observe that the reduction in alveolar height or width after alveolar preservation procedures was related to the thickness of the buccal bone plate [40,41].

It should be remembered that the healing process is also influenced by the patient’s hygiene [42]. Studies have shown that the use of propolis rinses contributes to a reduction in plaque and improved oral hygiene [43,44].

A positive correlation between optical bone density and primary implant stabilization has been confirmed by many researchers [45,46,47,48,49,50,51]. In the present study, our analysis proved that over the healing period, the optical density at the augmented site increased. Nevertheless, this process happened more rapidly for the xenograft group.

In their study, Khan et al. observed that allograft-augmented (DFDBA) sockets, in combination with platelet-rich fibrin, had higher bone optical density than naturally healing sockets [52], which could be due to the osteoinductive properties of DFDBA and PRF.

Similar studies were conducted by Loveless et al., where no statistically significant difference in bone optical density was found between alveoli filled with allografts (cortical FDBA, Straumann^®^ AlloGraft) and those not subjected to augmentation [53].

These results suggest that osteoinductive capacity may play a role in changes in bone optical density and requires further investigation. In our study, the xenograft group was characterized by a higher bone optical density, which may be due to a higher residual graft content rather than the osteoinductive capacity of the materials.

Elbanna et al. showed a statistically insignificant tendency towards a decrease in the bone optical density of sockets filled with xenogenic materials (cancellous DBBM, Inno Oss B^®^) mixed with PRF [54]. We did not use PRF for alveolar ridge preservation, which could explain the difference in our results.

The use of an alveolar preservation procedure causes changes in the tissue structure of the newly formed bone, such as the presence of residual biomaterial particles [55]. However, studies have not shown any differences in the primary stabilization of implants placed in the augmented area and those that heal naturally during the two-stage implantation procedure [56,57,58,59,60].

## 5. Conclusions

Alveoli filled with allogeneic material showed a statistically significantly lower reduction in alveolar buccal bone height compared to alveoli filled with xenografts. In contrast, alveoli filled with xenografts showed a statistically significantly higher optical density after the healing period. In addition, our histological analysis suggested a faster remodeling of the allografts into native bone. However, this should be interpreted with caution due to a possible discrepancy between the trephine biopsy area and the area filled with augmentation material.

## Figures and Tables

**Table 1:** Comparison of alveolar ridge width and height alterations, in millimeters, after 6 months of healing.

Clinical Parameter	Allograft		Xenograft		
	Mean (Sd.)	Median (IQR)	Mean (Sd.)	Median (IQR)	*p*-Value
Horizontal width alteration coronally	1.08 (0.61)	0.97 (0.60)	1.08 (0.68)	0.90 (0.68)	1.00
Horizontal width alteration midpoint	0.79 (0.25)	0.97 (0.45)	0.63 (0.47)	0.60 (0.60)	0.56
Horizontal width alteration apically	0.68 (0.35)	0.76 (0.44)	0.47 (0.32)	0.45 (0.30)	0.40
Vertical height alteration buccally	0.52 (0.22)	0.59 (0.29)	1.32 (0.45)	1.41 (0.45)	0.01 *
Vertical height alteration palatally	0.45 (0.44)	0.46 (0.61)	0.76 (0.25)	0.72 (0.23)	0.25

* Statistically significant.

**Table 2:** Mean optical bone density levels in Hounsfield units.

Material	1 Week		6 Months		
	Mean	Sd.	Mean	Sd.	*p*-Value
Allograft	1341.40	119.23	1499.94	249.70	0.28
Xenograft	1288.58	139.98	1881.08	262.40	0.004 *

* Statistically significant.

## Data Availability

The original contributions presented in the study are included in the article, further inquiries can be directed to the corresponding author.
